# Use of Contraceptive among Postpartum Women of a Municipality: A Descriptive Cross-sectional Study

**DOI:** 10.31729/jnma.5596

**Published:** 2021-07-31

**Authors:** Mukta Singh Bhandari, Suresh Kayastha

**Affiliations:** 1 Department of Community Medicine, Kathmandu University School of Medical Sciences, Dhulikhel, Kavrepalanchok, Nepal; 2 Department of Obstetrics and Gynaecology, Nepal Korea Friendship Municipality Hospital, Balkumari, Thimi, Bhaktapur, Nepal

**Keywords:** *contraception*, *contraceptive devices*, *postpartum period*

## Abstract

**Introduction:**

Contraception plays a key role in preventing unwanted births. It also decreases the pregnancy and childbirth-related morbidities and mortalities, but many women don't use contraception particularly in the postpartum period. The main objective of this study was to find out the prevalence of contraception use among postpartum women in a municipality.

**Methods:**

A descriptive cross-sectional study was conducted among married women of reproductive age in Dhulikhel municipality of Kavrepalanchok from January to September 2019. Ethical approval was obtained from Institutional Review Committee and permission was taken from Dhulikhel Municipality before the study. The sample size was 332. A convenience sampling method was used. A semi-structured questionnaire pre-tested in Panauti municipality was used. Data entry and analysis were done using Statistical Package for Social Sciences version 20. Point estimate at 95% Confidence Interval was calculated for descriptive analysis.

**Results:**

Out of total 332 women, 146 (40%) (34.73-45.26 at 95% Confidence Interval) used postpartum contraception. Injectable/Depot was used by 61 (42%) women. Total 97 (52%) of the non-users intended to use contraception in the future. The most common reason for contraception use was women not wanting the next child soon 91 (62%) and reason for non-use was feeling contraception as unnecessary 73 (39%).

**Conclusions:**

The use of postpartum contraception was poor, and only half of the non-users intended to use contraception in the future. Thus, contraception use should be encouraged during all possible contact times, and counselling should be made universal to improve postpartum contraception services' uptake.

## INTRODUCTION

Contraceptive leads to reduction in unplanned pregnancies and also improves maternal and child wellbeing. Contraception also helps to empower women by giving more choice over fertility and gives opportunities for education and career development.^1-5^

Despite many programs of Nepal focusing on family planning, 24% of women have unmet need for contraception and 21% of births take place before the recommended twenty four months interval.^6^ Also, very less information on postpartum contraception use is known about Dhulikhel municipality.

Thus, this study aims to find out the prevalence of postpartum contraception use among women living in Dhulikhel municipality of Kavrepalanchok.

## METHODS

A descriptive cross-sectional study was done at Dhulikhel municipality of Kavrepalanchok district of Nepal. The duration of study was from January 2019 to September 2019. Ethical approval was taken from Institutional Review Committee of Dhulikhel Hospital (Reference number 123/18) and permission to conduct study was taken from Dhulikhel municipality (Reference number 221). After explaining the objective of the study in detail, verbal consent was taken from each respondent. Women below the age of 49 years who had given birth within 6 weeks up to three years prior to the survey were included in the study. However, those women having still birth, neonatal or infant mortality or having any contraindication for contraceptive use were excluded from the study.

The sample size was calculated using the given formula.

n = Z^2^ × p × q / e^2^

  = (1.96)^2^ × (0.27) × (1-0.27) / (0.05)^2^

  = 302

Where,

n = the sample size
Z = 1.96 at 95% Confidence Interval
p = prevalence of postpartum contraception use, 27%^7^
q = 1-p
e = margin of error, 5%

After taking the non-response rate of 10% the final sample size was 322.

Five enumerators from medical and public health background were oriented before data collection. Data was collected using semi-structured questionnaire which was pre-tested at ward number five of Panauti municipality before the survey. Necessary modifications were done in the questionnaire before the actual study.

There are 12 wards in Dhulikhel municipality. Out of 12 wards, six wards i.e., ward number 1, 3, 4, 7, 9 and 11 were selected by probability proportion to size method. Household was considered as one unit and number of households in each ward were selected by proportion which gave the sample size of 38, 46, 67, 59, 56 and 66 in the above wards respectively. As many of the households didn't have eligible women and many households were empty during preliminary survey, convenience sampling method was used for the final data collection. One woman from each household was approached and if more than one eligible woman were present in a household, then the one with youngest child was selected. Households were taken until sample size was fulfilled.

A descriptive analysis of socio-demographic variables was done using mean, frequency, percentage and standard deviation. Data entry and analysis was done in Statistical Package for Social Sciences (SPSS) version 20.

## RESULTS

Out of total 332 women, 146 (44%) were using postpartum contraception at present which included all forms of contraception i.e., modern or natural methods.

The mean age of women was 26.21 ±4.27 years (Mean±SD). Total 260 (78%) women were Hindu and Janajati 172 (52%) by caste. Total 167 (50%) women had education up to SLC and 173 (52%) were home makers. Most of the women 180 (54%) lived in joint family and 299 (90%) had mass media exposure regarding contraceptive methods. Total 310 (93%) women had delivered in a health facility and 246 (74%) had normal delivery (Table 1).

**Table 1:** Socio-demographic profile of respondents.

Socio-demographic variables		n (%)
Religion	Hindu	260 (78)
	Buddhist	51 (15)
	Christian and others	21 (7)
Caste	Chhetri	69 (21)
	Brahmin	64 (19)
	Janajati	172 (52)
	Others	27 (8)
Education	Illiterate	13 (4)
	Literate	9 (3)
	Up to SLC	167 (50)
	Above SLC	143 (43)
Occupation of women	Farming	50 (15)
	Job	48 (14)
	Home maker	173 (52)
	Business	42 (13)
	Student and others	19 (6)
Type of family	Nuclear	152 (46)
	Joint	180 (54)
Mass media exposure	Yes	299 (90)
	No	33 (10)
Place of last birth	Health facility	310 (93)
	Home	22 (7)
Type of birth	Normal	246 (74)
	Cesarean section	86 (26)

Total 260 (78%) women have had resumption of menorrhea and the mean duration of amenorrhea was 5.9 ±3.77 months (Mean±SD). Out of 260 women who had resumption of menorrhea, 43 (16.5%) had resumption in less than three months, 93 (36%) in 3 to 6 months while 124 (48%) had resumption in greater than 6 months duration.

Around 41 (12%) women gave history of ever having unwanted pregnancy but none of them responded to have aborted the unwanted conception.

Total 117 (35%) women reported to have used any contraceptive method in the past and out of them, 49 (42%) reported to have had adverse effect with 20 women (41%) experiencing bleeding problem, 15 (31%) irregular cycle, 8 (16%) weight gain and 5 (12%) others which included weight loss 2 (4%), amenorrhea 2 (4%) and pain abdomen 1 (3%).

**Figure 1:**
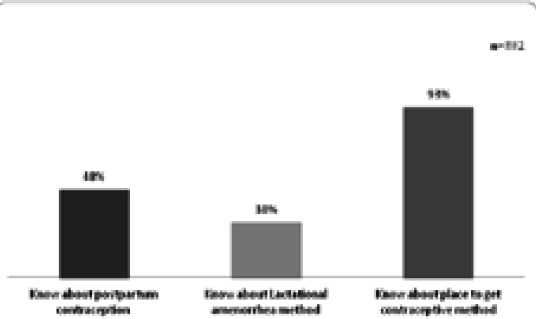
Knowledge about postpartum contraceptive method.

Out of total 332 women, 158 (48%) said that they know about postpartum contraceptive methods but only 119 (75%) out of them could correctly list at least two names of postpartum contraceptive methods (Figure 1).

Among 310 women who had given birth in a health facility, 237 (76%) received counseling regarding postpartum contraception while only 177 (57%) received counseling regarding adverse effects of contraceptive methods following delivery. Total 150 (63%) women reported having being counseled by nurses, 77 (32%) by doctors and 10 (5%) by nursing students. Likewise, only 100 (30%) women out of 332 reported to have received counseling regarding postpartum contraception during immunization of their child.

**Figure 2:**
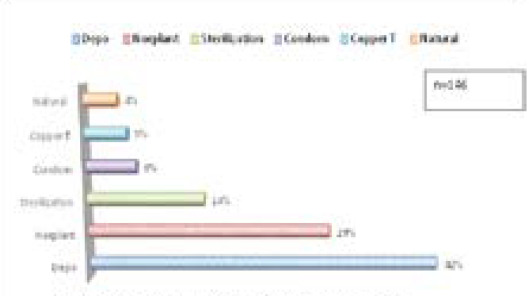
Methods used for postpartum contraception.

Out of 332 women, total 146 (44%) women reported to be using any form of postpartum contraception and most commonly reported method of contraception was Depot 61 (42%) while use of long acting reversible contraceptive method (LARC) was 50 (34%) (Figure 2).

Among contraceptive users, 91 (62%) women said that the reason for using contraception was not wanting another child soon while 4 (3%) said that they were advised by health worker and one (1%) said that she was advised by family member. Total 219 (66%) women said that decision of contraceptive use is jointly made by them along with husband while 63 (19%) reported making decision by themselves and 50 (15%) by husband.

**Figure 3:**
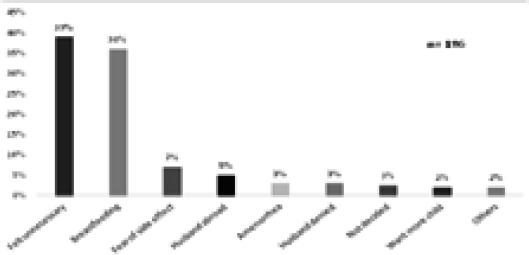
Reason for non-use of postpartum contraception.

Out of 332 women, 186 (56.02%) were non-users and 73 (39%) women among them stated using contraception as being unnecessary while 4 (2%) gave other reasons which were unavailability of intended method and current pregnancy (Figure 3).

Out of 186 non-users, 102 (54.83%) women didn't use contraception but also didn't want any child soon due to various reasons thus giving the unmet need of 57 (31%) among study group.

Out of 186 postpartum contraception non-users, 97 (52%) intended to use some form of contraception in the future and the contraceptive of choice were Norplant, sterilization and Depot reported by 20 (20%), 13 (13%), and 13 (13%) women respectively while 41 (42%) women said that they don't know what method they intended to use.

## DISCUSSION

The use of postpartum contraception in this study was poor and only some women had intention to use contraception in the future. Although counseling about contraception was done, women were not informed about the side effects of contraceptive methods.

The result of this study indicates poor utilization of postpartum contraception which is in line with studies done in Nepal^8,9^ and also studies from Bangladesh^10^ and Ethiopia^11-13^ showing utilization below 50%. But higher uses of postpartum contraception were found in Kenya^14^ (86%) and Liberia^15^ (77%) which may be due to more acceptability and accessibility to contraceptive services in those places.

The most common method of contraception used by women in this study was found to be injectable method or Depot which is similar to many studies done in Nepal,^9,16,17^ Ethiopia,^11–13,18^ Kenya^14^ which may be due to the easy accessibility, availability, less effect on lactation and ease of administration.

This study found that only around half of the postpartum contraception non-users intended to use any form of contraception in the future. This finding is consistent with findings from studies Nepal^9,16,17^ but very high intention to use was found in other parts of the world like Bangladesh^10^ Ethiopia^19^ and Ohio.^20^ The difference in socio-demographic structure, awareness, availability of services might have affected the intention of women to use postpartum contraception in the future.

Women who knew about postpartum contraception was below 50% which is similar to study from Kailali^8^ (42%) but higher proportion of women knew about it in Liberia^15^ (79%) and Indonesia (77%).^21^

Our study showed that current non-users of contraception intended to use long term method or permanent method compared to those who were already using any method of contraception which is consistent to findings from Ohio^20^ and Nigeria.^22^

Most common reason stated for not using any postpartum contraception in this study were feeling it as unnecessary and amenorrhea which were also seen in study done in Kailali,^8^ Ethiopia,^10–13^ and Egypt.^23^

Counseling regarding postpartum contraception was received by 3/4th of the women which is similar with other studies from Nepal.^9,16^ But the Nepal Demographic Health Survey showed 13% counseling in the postpartum period in the country, which might be due to the difference between service provider and coverage between different sectors.^6^ As the main service provider in our study was tertiary level teaching hospital, the service coverage might have been greater than the national data.

This study had certain limitations. Being a crosssectional study, the study could not explore more about the women's experience and reasons for utilization or non-utilization of postpartum contraception. Due to lack of feasibility and cost, the study participants couldn't be randomly selected at household level. As the study was done in different wards of Dhulikhel municipality, it doesn't represent whole of Kavrepalanchok or Nepal.

## CONCLUSIONS

The use of postpartum contraception was poor and knowledge about postpartum contraceptive devices was also not good. Women didn't feel the need of contraception; thus, counseling should be made universal at all contact periods starting from pregnancy.
